# Ketogenic diet feeding improves aerobic metabolism property in extensor digitorum longus muscle of sedentary male rats

**DOI:** 10.1371/journal.pone.0241382

**Published:** 2020-10-30

**Authors:** Yuji Ogura, Chiaki Kakehashi, Toshinori Yoshihara, Mitsutoshi Kurosaka, Ryo Kakigi, Kazuhiko Higashida, Sei-Etsu Fujiwara, Tatsuo Akema, Toshiya Funabashi

**Affiliations:** 1 Department of Physiology, St. Marianna University of School of Medicine, Miyamae-ku, Kawasaki, Japan; 2 Graduate School of Health and Sports Science, Juntendo University, Inzai, Chiba, Japan; 3 Faculty of Management & Information Science, Josai International University, Togane, Chiba, Japan; 4 Department of Nutrition, University of Shiga Prefecture, Hikone, Shiga, Japan; University of Minnesota Medical School, UNITED STATES

## Abstract

Recent studies of the ketogenic diet, an extremely high-fat diet with extremely low carbohydrates, suggest that it changes the energy metabolism properties of skeletal muscle. However, ketogenic diet effects on muscle metabolic characteristics are diverse and sometimes countervailing. Furthermore, ketogenic diet effects on skeletal muscle performance are unknown. After male Wistar rats (8 weeks of age) were assigned randomly to a control group (CON) and a ketogenic diet group (KD), they were fed for 4 weeks respectively with a control diet (10% fat, 10% protein, 80% carbohydrate) and a ketogenic diet (90% fat, 10% protein, 0% carbohydrate). After the 4-week feeding period, the extensor digitorum longus (EDL) muscle was evaluated ex vivo for twitch force, tetanic force, and fatigue. We also analyzed the myosin heavy chain composition, protein expression of metabolic enzymes and regulatory factors, and citrate synthase activity. No significant difference was found between CON and KD in twitch or tetanic forces or muscle fatigue. However, the KD citrate synthase activity and the protein expression of Sema3A, citrate synthase, succinate dehydrogenase, cytochrome c oxidase subunit 4, and 3-hydroxyacyl-CoA dehydrogenase were significantly higher than those of CON. Moreover, a myosin heavy chain shift occurred from type IIb to IIx in KD. These results demonstrated that the 4-week ketogenic diet improves skeletal muscle aerobic capacity without obstructing muscle contractile function in sedentary male rats and suggest involvement of Sema3A in the myosin heavy chain shift of EDL muscle.

## Introduction

The ketogenic diet, a high-fat diet with low or absent carbohydrate, mimics the fasting state of the body [1]. This diet increases ketone production from the liver and shifts energy sources to fat instead of carbohydrate [2–6]. The generated ketone is used eventually as an energy substrate in different organs such as the brain and skeletal muscles.

Earlier studies have been undertaken to elucidate therapeutic effects of a ketogenic diet on the central nervous system. For example, a ketogenic diet is regarded as an alternative treatment to anticonvulsants for a patient with pharmacoresistant or medication-intolerant epilepsy [7–10]. Although the precise mechanism remains unknown, this effect is reportedly associated with a change in the vesicular glutamate transporter activity and quantal size in the vesicle at hippocampal synapses [11]. Furthermore, proposals for clinical application of a ketogenic diet have targeted amelioration of different diseases including hyperglycemia in diabetes [12–15], cardiomyopathy with glycogen storage disease type III [16, 17], cancer development [4, 18], and age-related brain dysfunction [19].

Skeletal muscle, in addition to supporting locomotion and respiration, is an important organ for whole-body metabolism [3, 20]. Recent reports of the relevant literature have described effects of a ketogenic diet on rodent skeletal muscle metabolic characteristics. Kephart et al. found that rats fed a ketogenic diet for 8 months exhibited decreased mitochondrial respiration capacity of gastrocnemius muscle [21]. Schnyder et al. reported that mice fed a ketogenic diet for 12 weeks showed lowered mitochondrial ATP production in gastrocnemius muscle [5]. In contrast to these reports of other studies, Shimizu et al. reported that mice fed a ketogenic diet for 12 weeks showed upregulated transcript levels of fatty acid oxidation in gastrocnemius muscle [22]. Therefore, the effect of ketogenic diet on muscle metabolic characteristics is contradictory. Furthermore, it remains unknown whether a ketogenic diet can affect skeletal muscle performance, which is associated with muscle metabolic characteristics.

Therefore, this study was conducted to clarify ketogenic diet effects on muscle force production and fatigability in isolated rat extensor digitorum longus (EDL) muscle. Additionally, to clarify biochemical and metabolic adaptation to the ketogenic diet, this study investigated muscle fiber types and metabolic enzymes for glucose and fat utilization.

## Materials and methods

### Ethical approval

The Animal Use Committee at St. Marianna University School of Medicine approved all experiment procedures (Protocol Number: 1006015). They were conducted according to guiding principles of the Physiological Society of Japan for the care and use of animals in the field of physiological sciences.

### Animal care and experiment design

From a licensed laboratory animal vendor (Japan Charles River Laboratories, Inc., Yokohama, Japan), 32 male Wistar rats (7 weeks old) were obtained. Upon arrival at our institution, all animals were provided with standard rodent food and water *ad libitum*. They were housed in an environmentally controlled room (24 ± 1°C, 55 ± 5% relative humidity: 12:12 h light-dark photoperiods (lights off 1800–0600)). Following a week of acclimation, the animals were assigned to a control diet group (CON, *n* = 16) or to a ketogenic diet group (KD, *n* = 16). The animals were then kept for four weeks with feeding of a specialized diet and water *ad libitum*. The body mass of each rat was recorded every week.

The animal diet used for this study was identical to that described for our earlier study [23]. The ketogenic diet composition was 10% protein and 90% fat (#D10070801; Research Diet Inc., New Brunswick, NJ, USA). The corresponding control diet, recommended by the supplier, included 10% protein, 10% fat, and 80% carbohydrate (#D10070802; Research Diet Inc.). Details of ingredients used for the respective diets are shown in Table 1.

**Table 1:** Compositions of control and ketogenic diets.

Ingredient	Control diet		Ketogenic diet	
	%grams	%kcal	%grams	%kcal
Casein, 80 Mesh	9.46	9.85	16.51	9.85
L-Cysteine	0.14	0.15	0.25	0.15
Corn starch	35.11	36.56	0.00	0.00
Maltodextrin 10	3.31	3.45	0.00	0.00
Sucrose	38.43	40.01	0.00	0.00
Cellulose.3 BW200	4.73	0.00	8.26	0.00
Soybean Oil	2.37	5.54	4.13	5.54
Cocoa butter	1.89	4.43	62.92	84.46
Mineral Mix, S10026	0.95	0.00	1.65	0.00
Dicalcium phosphate	1.23	0.00	2.15	0.00
Calcium carbonate	0.52	0.00	0.91	0.00
Potassium citrate	1.56	0.00	2.72	0.00
Vitamin mix, V10001C, 10X	0.09	0.00	0.17	0.00
Choline bitartrate	0.19	0.00	0.33	0.00
FD&C Yellow Dye #5	0.002	0.000	0.004	0.000
FD&C Red Dye #40	0.000	0.000	0.004	0.000
FD&C Blue Dye #1	0.002	0.000	0.000	0.000
Total	100	100	100	100

Each diet was purchased from Research Diet Inc.

The ketogenic diet was stocked at -80°C and was changed to provide fresh food every second day. After four weeks, the animals were anesthetized using isoflurane. Then a blood sample was collected from the abdominal vein to measure the blood β-hydroxybutyric acid concentration as an index of blood ketone body. Commercially available test strips were used (Precision Xceed; Abbott Japan Co. Ltd., Minato-ku, Tokyo, Japan). For some animals, the blood glucose was also measured using test strips (Abbott Japan Co. Ltd.). Then the EDL muscle was removed, weighed, and frozen for subsequent analyses. For this study, EDL muscle was selected because it is a fast-twitch fiber muscle similar to the gastrocnemius and quadriceps femoris muscles, which have been used often in earlier ketogenic diet studies [22, 24–26]. Also, EDL muscle is suitable to isolate with tendon-to-tendon manner for performing muscle contraction experiments.

The animals were euthanized by removal of the heart after EDL removal. The removed muscle was used to measure muscle glycogen, muscle fiber types, and muscle protein expression (CON, *n* = 5; KD, *n* = 5). The protein expression included representative metabolic enzymes for glucose and fat utilization, myosin heavy chain (MyHC) type II isoforms, and potent regulatory factors for muscle oxidative metabolism and muscle fiber type. The remaining animals were used for muscle contraction analyses: twitch and tetanic tension (CON, *n* = 6; KD, *n* = 5), and muscle fatigue (CON, *n* = 5; KD, *n* = 6).

### Sample preparation for biochemical analyses

The frozen EDL muscles were thawed and homogenized (1:9 w/v) in ice-cold buffer (50 mM Tris-HCl, 200 mM NaCl, 50 mM NaF, 0.3% NP-40, pH 8.0) with protease and phosphatase inhibitors (Nacalai Tesque Inc., Chukyo-ku, Kyoto, Japan). After the muscle homogenates were centrifuged at 1000 × *g* for 20 min at 4°C, the supernatant was collected. Total protein concentrations of the supernatant were estimated using BCA protein assay reagent (Life Technologies Japan Ltd., Minato-ku, Tokyo) with bovine serum albumin as a standard using a microplate reader (Multiskan MS; Life Technologies Inc.). A part of the supernatant was used directly for CS activity measurements. The remainder of the supernatant was denatured with Laemmli sample buffer at 95°C for 5 min for one-dimensional sodium dodecyl sulfate polyacrylamide gel electrophoresis (SDS-PAGE).

Insoluble sediments of the homogenate described above were re-suspended (1:99 w/v) using MyHC sample buffer (30% glycerol, 5% ß-3-mercapto-1,2-propanediol, 2.3% SDS, 0.05% bromophenol blue, 62.5 mM Tris-HCl, pH 6.8). Then they were denatured at 65°C for 15 min.

### Western blotting

Western blotting was applied to examine muscle metabolic adaptation of EDL. After the sample was separated using standard SDS-PAGE, it was transferred to a PVDF membrane [27]. The membrane was then stained with Ponceau-S solution to estimate the total transferred protein (TTP). The membrane was blocked with a blocking reagent (Blocking One; Nacalai Tesque Inc.) and was incubated with the primary antibodies listed below for the blocking solution. After several repetitions of washing, the membranes were incubated with HRP-linked anti-mouse IgG or anti-rabbit IgG secondary antibody (1:10000, Cell Signaling Technology Inc., CST, Danvers, MA, USA), or anti-mouse IgM Alexa fluor 488-linked secondary antibody (1:2000, Life Technologies Japan Ltd.) for 1 h at 25°C. The membrane was then visualized using enhanced chemiluminescence reagent (Clarity Max; Bio-Rad Laboratories Inc., Hercules, CA, USA) or a blue LED using an imaging system (LAS-3000; GE Healthcare Japan, Hino, Tokyo, Japan). Densitometry analysis was conducted using analytical software (Li-Cor Inc., Lincoln, NE, USA). Then the data were normalized by TTP for each sample [28].

Primary antibodies used for this study were the following: anti-phosphofructokinase (PFK, 1:1000, sc-166722), anti-CS (1:1000, sc-390693), anti-succinate dehydrogenase (SDH, 1:1000, sc-166947), anti-3-hydroxyacyl-CoA dehydrogenase (HAD, 10:1000, sc-376525) anti-peroxisome proliferator-activated receptor-alpha (PPAR-α, 1:1000, sc-398394), and anti-semaphorin 3A (Sema3A, 1:1000, sc-74554) from Santa Cruz Biotechnology, Inc. (Dallas, TX, USA); anti-cytochrome c oxidase-IV (COX-IV, 1:2000, #5274) from Cell Signaling Technology Japan, K.K. (Chiyoda-ku, Tokyo, Japan); anti- MyHC type IIa (1:200, SC-71), anti-MyHC type IId (1:200, 6H1), anti-MyHC type IIb (1:200, BF-F3) from The Developmental Studies Hybridoma Bank (Iowa City, IA, USA); anti-peroxisome proliferator-activated receptor gamma coactivator 1-alpha (PGC-1α, 1:2000, T1202) from Merck Millipore (Burlington, MA, USA); and anti-myogenic enhancer factor 2C (Mef2C, 1:1000 NBP2-17260) from Novus Biologicals, LLC (Centennial, CO, USA).

To estimate glycolysis capacity, PFK was analyzed [29]. Also, CS, SDH, and COX-IV were analyzed to estimate oxidative capacity [30–32]. To estimate beta-oxidation capacity, HAD was analyzed [33]. As major molecules to regulate muscle oxidative metabolism, PGC-1α, PPAR-α, and Mef2C were measured [5, 34–40]. Also, Sema3A was analyzed as a potential mechanism for muscle fiber type regulation [41].

### Analysis of MyHC composition

The MyHC composition was found using glycerol–SDS-PAGE, as reported for our earlier studies [31, 42]. Briefly, the prepared protein samples were applied in duplicate to the SDS-PAGE gel: stack– 4% acrylamide, 34.7% glycerol, 125 mM Tris-HCl pH 6.8; separation– 8% acrylamide, 33.3% glycerol, 375 mM Tris-HCl, pH 8.3. Electrophoresis was started at 60 V with stacking gel at 8°C. The voltage was set to 150 V and run for 18 h at 8°C when the tracking dye had entered the separating gel completely. After separation, each gel was stained with Coomassie brilliant blue (Biosafe G250; Bio-Rad Laboratories Inc., Hercules, CA, USA). To ascertain the relative proportion of MyHC isoforms, each gel was scanned using a calibrated densitometer (ChemiDoc Touch; Bio-Rad Laboratories Inc., Hercules, CA, USA) and was examined using analytical software (Image Lab; Bio-Rad Laboratories Inc.).

### Citrate synthase (CS) activity

To estimate the mitochondrial content [32] of EDL muscles as described earlier, the CS activity was measured as described in a report of our early study [42]. The reaction mixture contained 100 mM Tris, 0.07% Triton X-100, 0.1 mM Ellman’s reagent (DTNB), 0.098 mM acetyl CoA, and 0.5 mM oxaloacetic acid at pH 8.3, and an appropriate volume of the supernatant prepared above [43]. Each measurement was taken spectrophotometrically (412 nm) in triplicate using a microplate reader (Multiskan MS; Life Technologies Inc.). The close two values were used for analyses. The activity was expressed as the specific activity corrected with the protein concentration of the supernatant (mM·min^-1^·mg protein^-1^).

### Muscle glycogen content

Unstimulated EDL muscle was used for these analyses [44]. After the muscle was powdered in liquid nitrogen, it was lysed in NaOH (1M) at 25°C for 5 min, followed by incubation at 85°C for 10 min. After HCl (6 M) was added (1:2), the mixture was incubated further at 85°C for 2 h. Finally, NaOH (4M) was added (1:2) to neutralize the lysate. The glucose concentration of the muscle lysate was measured using a commercially available kit (Glucose Wako C-II kit; Fujifilm Wako Pure Chemical Corp., Chuo-ku, Tokyo, Japan). The muscle glycogen content (mM) was calculated using the dilution factor of each lysate.

### Muscle force measurement

The muscle performance was measured by ex-vivo muscle contraction experimentation. After the removal of EDL muscle (tendon to tendon), the muscle was transferred into a Krebs–Henseleit solution (117 mM NaCl, 5.9 mM KCl, 1.2 mM NaH_2_PO_4_, 2.5 mM CaCl_2_, 1.2 mM MgCl_2_, 24.8 mM NaHCO_3_, 11.1 mM glucose, 25 μM D-tubocurarine chloride). The buffer was equivalented continuously to pH 7.4 with a 95% O_2_ and 5% CO_2_ gas mixture at 25°C. The EDL muscle was set vertically in a specialized organ bath with one end connected to a calibrated isometric force transducer (TB-611T; Nihon Kohden Corp., Shinjuku-ku, Tokyo, Japan) using bladed silk. After a 10 min equilibration period, the optimal EDL muscle length was found using a micrometer. The maximal voltage evoking a single twitch was also found using electrical field stimulation (SEN-3301; Nihon Kohden Corp.). Once the experimental condition was fixed, a twitch was evoked (1 Hz, 1 ms for the duration). After a 5 min resting period, tetanic contraction was evoked by field stimulation of 90 Hz, 300 ms for the duration with supramaximal stimulation voltage (1.5 fold voltage evoking the twitch). Each tetanic contraction was performed sequentially but separated by a 3 min recovery period. Muscle fatigue was measured in another subset of animals. The EDL muscle was prepared as described above: tetanic contraction was evoked at 60 Hz for 300 ms duration at 3 s intervals for 10 min. Data were recorded every minute.

The muscle force was amplified using a carrier amplifier (AP-621G; Nihon Kohden Corp.). Data were stored digitally in a computer via an A/D converter (USB-6008; National Instruments Japan Corp., Minato-ku, Tokyo, Japan) controlled by software (LabVIEW; National Instruments Japan Corp.). Muscle force was normalized by the total muscle cross-sectional area to ascertain the specific tension. The total muscle cross-section was estimated as [muscle mass (g)/(muscle length (cm) × 1.056)], where 1.056 is the muscle density (g·cm^-3^)[45, 46].

### Statistical analysis

Data are presented as means ± standard error of the mean (S.E.M.). Differences between CON and KD were analyzed using unpaired *t*-tests. Body mass and fatigue experiments were analyzed using two-way repeated measures ANOVA (diet and time). When interaction was found, Bonferroni multiple comparison tests were applied to evaluate the significance of differences between the CON and KD groups. Probability of *p* < .05 was inferred as significant. All statistical analyses were conducted using software (Prism 8.1.2; GraphPad Software Inc., CA, USA).

## Results

### Body mass, blood parameters, and muscle mass

**Fig 1:**
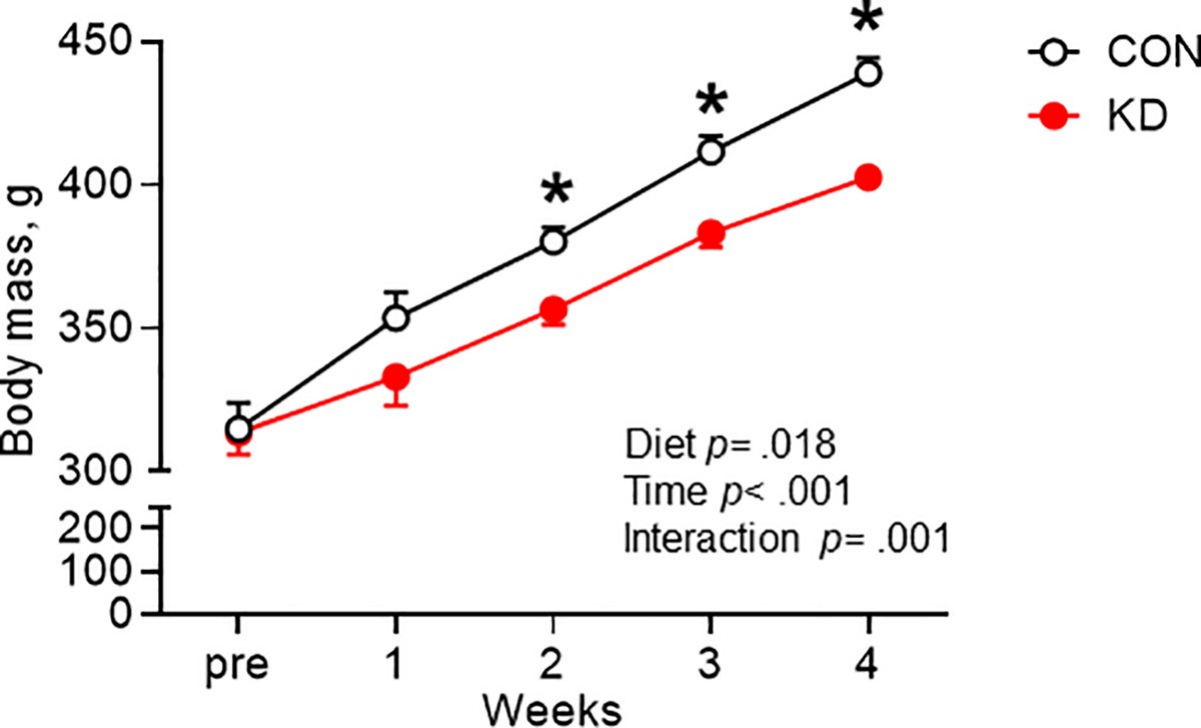
Change in body mass during the experimental period in CON (*n* = 16) and KD (*n* = 16). * Significant difference was found between CON and KD. Values are expressed as means ± S.E.M.

Fig 1 depicts the change in body mass during the 4-week experimental period. Two-way ANOVA revealed significant main effects of diet (*F*_1, 30_ = 6.239, *p* = .018) and time (*F*_1.253, 37.58_ = 367.8, *p* < .001). Significant interaction (diet and time) was found between groups (*F*_4, 120_ = 8.721, *p* < .001). Multiple comparison analysis revealed that the body mass of KD was significantly lower than that of CON at week 2 (*t* = 3.403, df = 30.21, *p* = .001), week 3 (*t* = 4.141, df = 31, *p* = .001), and week 4 (*t* = 5.817.42, df = 29.9, *p* < .001).

The blood β-hydroxybutyric acid concentration of KD was found to be significantly higher than that of CON (*t* = 12.42, df = 30, *p* < .0001; Table 2). The blood glucose concentration of KD was significantly lower than that of CON (*t* = 2.919, df = 8, *p* = .019; Table 2). No difference was found between CON and KD in EDL muscle mass after the experimental period (Table 2). However, as expected, the relative muscle mass of KD was significantly higher than that of CON (*t* = 2.180, df = 30, *p* = .037; Table 2) because the body weight of KD was significantly lower than that of CON.

**Table 2:** Blood β-hydroxybutyric acid concentration, blood glucose concentration, and EDL muscle mass in CON and KD animals after the 4-week experimentation period.

	CON	KD
Blood β-hydroxybutyric acid, mM	0.32 ± 0.03	1.71 ± 0.11*
Blood glucose, mM	9.8 ± 0.8	7.3 ± 0.4*
Muscle mass, mg	205 ± 4	198 ± 5
Relative muscle mass, mg·g^-1^	0.47 ± 0.01	0.49 ± 0.01*

CON (*n* = 16) and KD (*n* = 16) for the blood β-hydroxybutyric acid concentration. (*n* = 5) and KD (*n* = 5) for blood glucose concentration. CON (*n* = 16) and KD (*n* = 16) for muscle mass. * Significant difference was found between CON and KD. Values are expressed as means ± S.E.M.

### Muscle glycogen content and CS activity

No significant difference was found between CON and KD in muscle glycogen content (Table 3). The citrate synthase (CS) activity of KD was significantly higher than that of CON (*t* = 2.33, df = 8, *p* = .0479; Table 3).

**Table 3:** Glycogen content and CS activity in EDL muscle of CON and KD animals.

	CON	KD
Glycogen content, mM	30.0 ± 0.4	28.0 ± 2.8
CS activity, mM·min^-1^·mg protein^-1^	79 ± 9	103 ± 4*

CON (*n* = 6) and KD (*n* = 5) for glycogen content. CON (*n* = 5) and KD (*n* = 5) for CS activity. * Significant difference was found between CON and KD. Values are expressed as means ± S.E.M.

### Protein expression of metabolic enzymes

**Fig 2:**
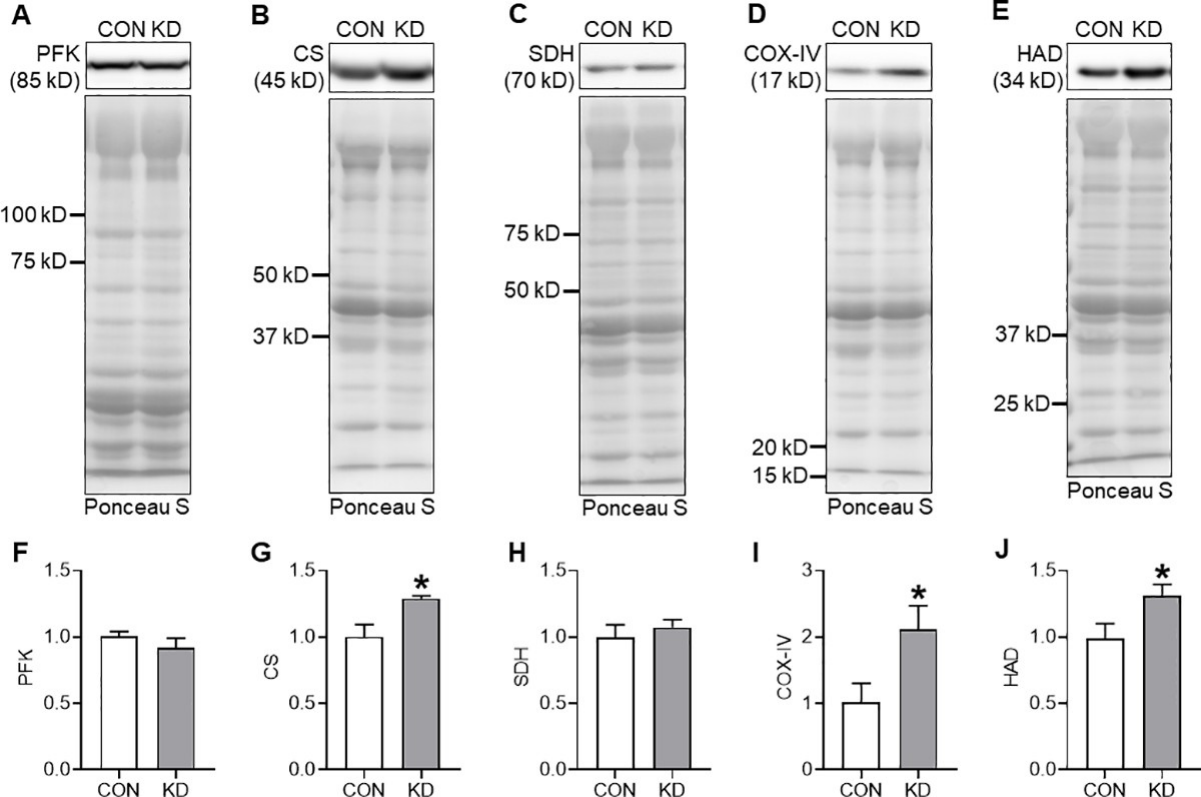
Protein expression of metabolic enzymes in CON (*n* = 5) and KD (*n* = 5). Representative images of the protein band and transferred protein of PFK (A), CS (B), SDH (C), COX-IV (D), and HAD (E). Protein expressions of PFK (F), CS (G), SDH (H), COX-IV (I), and HAD (J). * Significant difference was found between CON and KD. Values are expressed as means ± S.E.M.

Results of the protein expression of PFK, CS, SDH, COX-IV, and HAD enzymes are presented in Fig 2. Fig 2A–2E portray each representative protein band and the transferred proteins onto the membrane. No significant difference in the expression of PFK was found between CON and KD (Fig 2F). The expression of CS in KD was significantly higher than that in CON (*t* = 3.012, df = 8, *p* = .017; Fig 2G). No significant difference in the expression of SDH between CON and KD was found (Fig 2H). The expression of COX-IV in KD was significantly higher than that in CON (*t* = 2.428, df = 8, *p* = .041; Fig 2I). Moreover, the expression of HAD in KD was significantly higher than that in CON (*t* = 2.315, df = 8, *p* = .049; Fig 2J).

### MyHC

**Fig 3:**
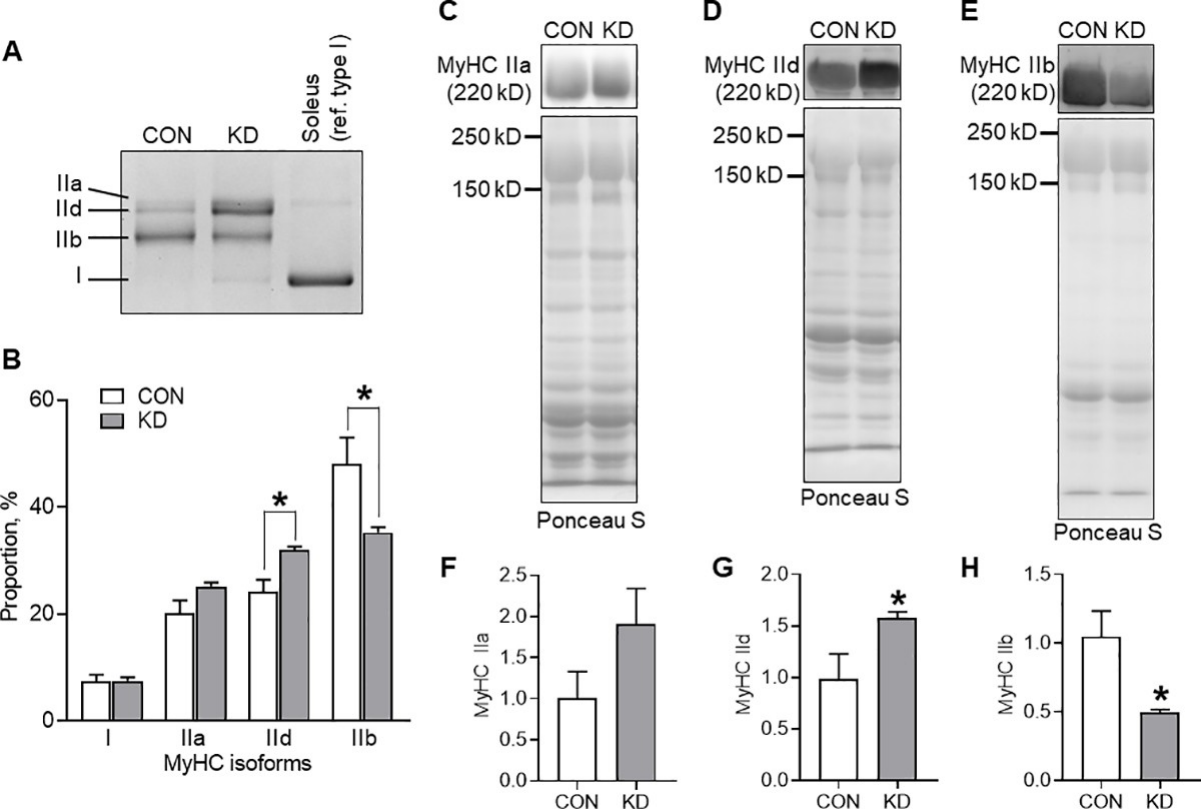
Proportion and the protein expression of MyHC isoforms in CON (*n* = 5) and KD (*n* = 5). (A) A representative image of the separation. Soleus was used for a reference of type I isoform. (B) Relative proportion of MyHC isoforms presented with representative images of the protein band and transferred protein of MyHC type IIa (C), IId (D), and IIb (E). Protein expressions of MyHC type IIa (F), IId (G), and IIb (H). * Significant difference was found between CON and KD. Values are expressed as means ± S.E.M.

Fig 3A presents a representative image of MyHC isoform separation. The quantitative results are presented in Fig 3B. No significant result was found for the proportion of type I and type IIa MyHC between CON and KD. However, the proportion of type IId in KD was significantly higher than that in CON (*t* = 3.336, df = 8, *p* = .010). By contrast, the proportion of type IIb in KD was significantly lower than that in CON (*t* = 2.613, df = 8, *p* = .031).

Fig 3C–3E exhibit a representative protein band and the transferred proteins onto the membrane in MyHC type IIa, IId, and IIb. No significant difference was found in the protein expression of MyHC type IIa between CON and KD (Fig 3F). However, the expression of MyHC type IId in KD was significantly higher than that in CON (*t* = 2.354, df = 8, *p* = .046; Fig 3G). The expression of MyHC type IIb in KD was significantly lower than that in CON (*t* = 2.936, df = 8, *p* = .019; Fig 3H).

### Protein expression of PGC-1α, PPAR-α, Mef2C, and Sema3A

**Fig 4:**
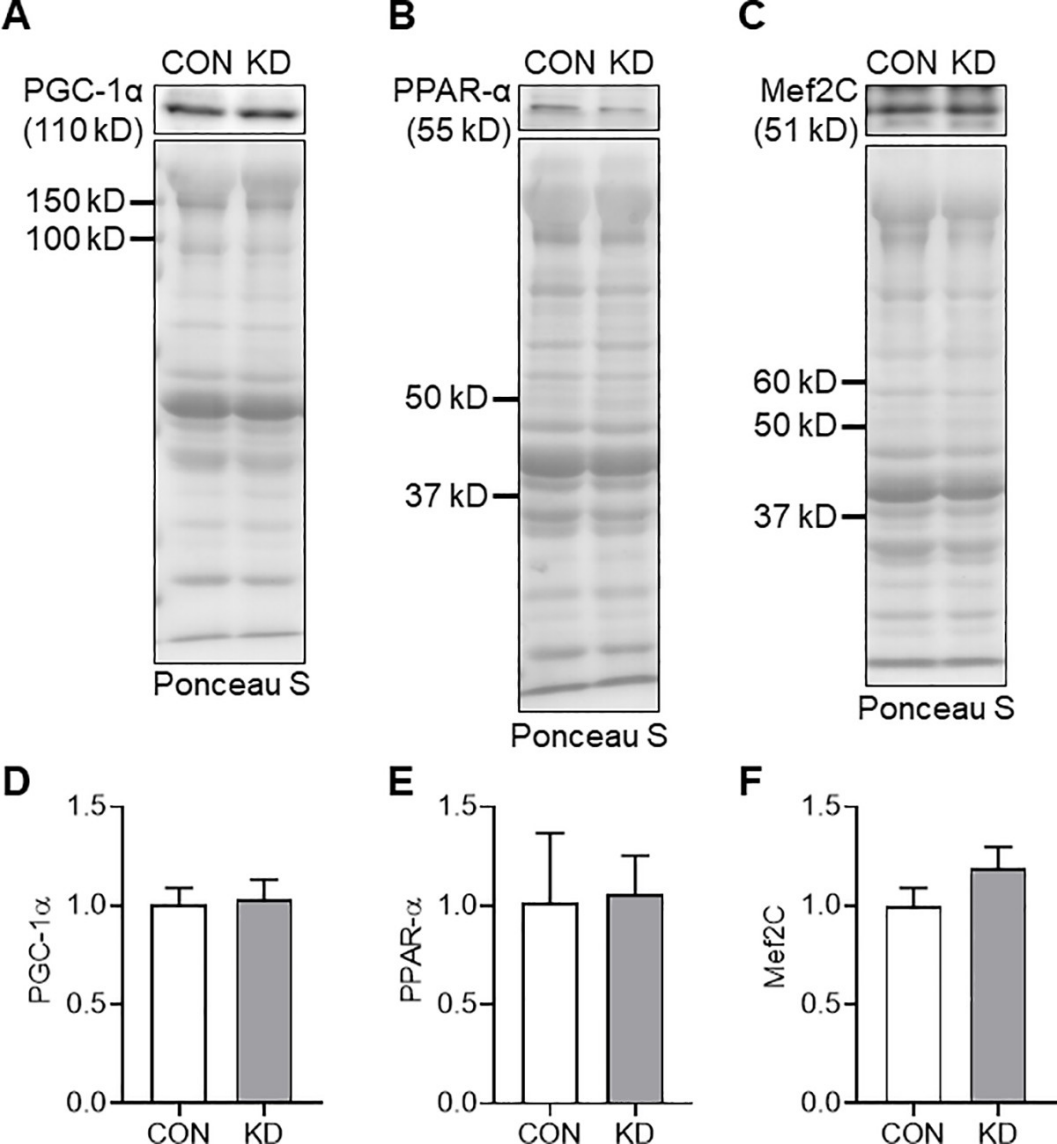
Protein expression of PGC-1α, PPAR-α, and Mef2C in CON (*n* = 5) and KD (*n* = 5). Representative images of the protein band and transferred protein of PGC-1α (A), PPAR-α (B), and Mef2C (C). Protein expressions of PGC-1α (D), PPAR-α (E), and Mef2C (F). Values are expressed as means ± S.E.M.

Because we found increased expression of CS, COX-IV, and HAD in addition to fast-to-slow shift of MyHC type II isoforms, we analyzed the expressions of PGC-1α, PPAR-α, Mef2C, and key proteins related to those changes as potential mechanisms (Fig 4). Fig 4A–4C show representative protein bands and the transferred proteins onto the membrane. However, no significant difference was found between CON and KD for the expressions of PGC-1α (Fig 4D), PPAR-α (Fig 4E), or Mef2C (Fig 4F).

**Fig 5:**
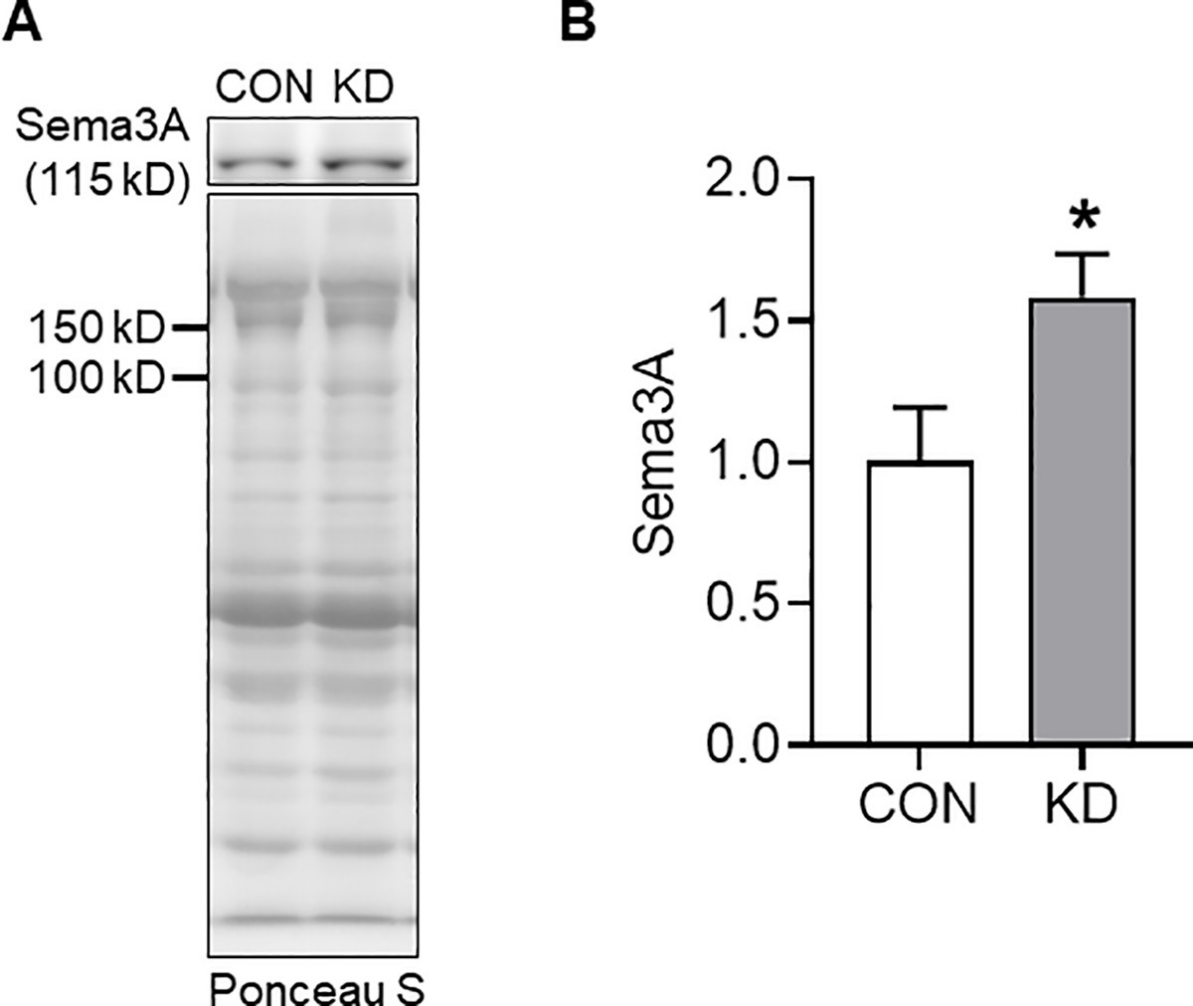
Protein expression of Sema3A in CON (*n* = 5) and KD (*n* = 5). Representative images of the protein band and transferred protein (A) The protein expression of Sema3A (B). *Significant difference was found between CON and KD. Values are expressed as means ± S.E.M.

The expression of Sema3A, which was found to regulate fast-twitch muscle fiber generation [41], in KD, was significantly higher than that in CON (*t* = 2.392, df = 8, *p* = .044; Fig 5A and 5B)

### Muscle force measurement

**Fig 6:**
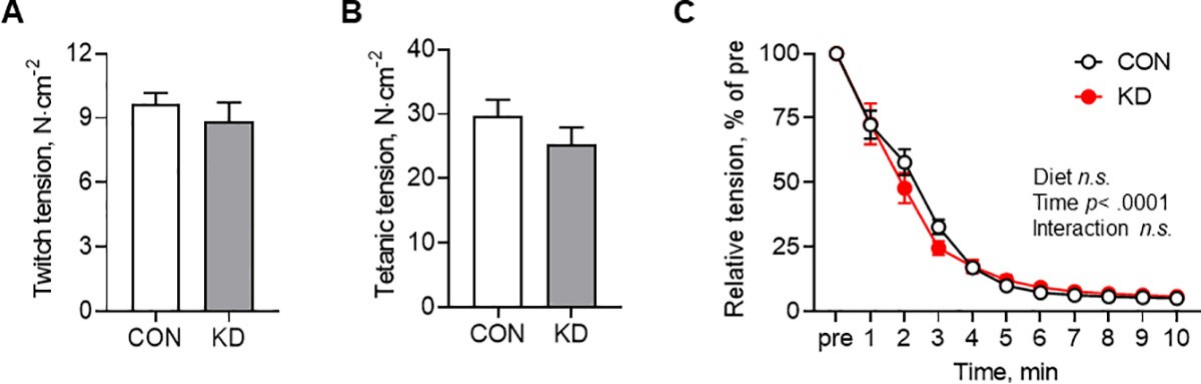
Muscle force of CON and KD. (A) Twitch tension of CON (*n* = 6) and KD (*n* = 5). Values are expressed as means ± S.E.M. (B) Tetanic tension under stimulation at 90 Hz, 300 ms duration in CON (*n* = 6) and KD (*n* = 5). Values are expressed as means ± S.E.M. (C) Fatigue experiment of CON (*n* = 5) and KD (*n* = 5). A main effect of time was found (*p* < .001). Values are expressed as means ± S.E.M.

Fig 6 presents the muscle force results. No difference was found between CON and KD in twitch (Fig 6A) or tetanic tension (Fig 6B). From fatigue experimentation (Fig 6C), two-way ANOVA was found to have a significant main effect of time (*F*_1.408, 12.67_ = 342.2, *p* < .001). However, significance was found neither for the main effect of diet nor for the interaction of diet and time.

## Discussion

This study was conducted to clarify ketogenic diet effects on skeletal muscle contractile function and biochemical characteristics in rats. Results indicate that four weeks of ketogenic diet feeding prevented increase in body mass with age, as demonstrated by earlier studies [2, 6, 23–26, 47, 48]. Results show that ketogenic diet feeding did not change muscle force production or fatigue resistance in isolated EDL muscle. Importantly, the ketogenic diet induced MyHC shift from type IIb to IIx. Moreover, this shift was accompanied by improved enzyme expression and activity for mitochondrial and fatty acid metabolism. Although the findings are limited to those for fast-twitch muscles, these results suggest that the ketogenic diet improves muscle aerobic energy metabolism without compromising muscle performance. Results also demonstrate that Sema3A might mediate MyHC shift in EDL.

Results show that the ketogenic diet increased CS activity and CS and COX-IV protein expression in EDL muscle: CS is an important enzyme in the tricarboxylic acid cycle in mitochondria; COX-IV is a mitochondrial enzyme that mediates the respiratory chain reaction. The respective expressions of these enzymes are indicators of mitochondrial content and oxidative capacity in skeletal muscle [32]. Consequently, our results suggest that a ketogenic diet improves oxidative capacity in skeletal muscle. However, these findings contradict those of studies that have shown lowered mitochondria function [5, 21] and decreased CS activity [21] by a ketogenic diet. In this regard, Parker et al. reported recently that a ketogenic diet followed for four weeks decreased CS activity in the red portion, but not in the white portion of gastrocnemius muscles in rats [25]. This finding suggests that mitochondrial enzyme adaptation to ketogenic diet depends on the muscle fiber type. In addition, EDL muscle has different proportions of muscle fiber types from those of gastrocnemius muscle [42, 49] used in earlier studies [5, 21, 25]. Although our results indicate a positive role of ketogenic diet on mitochondria, a study examining muscle fiber types must be conducted to ascertain ketogenic diet effects on those enzymes in skeletal muscle.

Results obtained for muscle glycogen, an important energy source in a fast-twitch muscle such as EDL, indicate no change in glycogen content after four weeks of ketogenic diet feeding. By contrast, Shimizu et al. reported that four weeks of ketogenic diet decreased muscle glycogen content in mice [22]. One difference between the results reported herein and those reported by Shimizu et al. [22] is adaptation of the fat oxidation enzyme. Although Shimizu et al. found no change in HAD gene expression [22], results of this study show that the ketogenic diet increased HAD protein expression, suggesting improved fat oxidation capacity of KD animals. Efficient fat utilization capacity is helpful to store muscle glycogen content. Indeed, keto-adapted skeletal muscle showed enhanced fat oxidation capacity, but comparable muscle glycogen content to that of the high-carbohydrate diet condition in humans [50]. Therefore, we speculate that improved fat oxidation capacity engenders reserve muscle glycogen content in our KD animals. Regarding glycogen usage, results show that the ketogenic diet did not change the expression of PFK, a rate-limiting enzyme of glycolysis, in EDL muscle. Increased energy demand from glycolysis reportedly improves PFK activity in skeletal muscle [29, 42, 51]. Consequently, our results suggest that the energy demand of glycolysis in EDL muscle during the four-week ketogenic diet is similar to that obtained from the control diet.

The skeletal muscle fiber type is determined by the MyHC isoform. An important finding of this study is that the four-week ketogenic diet induced a fast-to-slow shift among type II MyHC isoforms in EDL muscle. Slower type II fiber is generally accepted as showing higher aerobic metabolic capacity than faster type II fibers [49, 52]. Moreover, exercise training-induced increase in type IIa and IIx isoforms and decrease in type IIb isoform are associated with improved muscle aerobic capacity [31, 42, 53]. Therefore, our results indicate that ketogenic diet improves muscle aerobic capacity in the view of muscle fiber type transformation. Although the finding remains controversial, a ketogenic diet reportedly ameliorates hyperglycemia in a diabetic rat model [13, 54]. A recent study using single fiber analysis revealed that insulin-stimulated glucose uptake was higher in order of type I and IIa, IIx, and IIb fibers in rats [55]. Consequently, our results indicate that such a favorable effect on blood glucose regulation by ketogenic diet [13, 54] might be associated partially with the fast-to-slow type II fiber shift in skeletal muscle.

In skeletal muscles, PGC-1α [5, 34–36], PPAR-α [36, 37], and Mef2C [34, 38–40] are representative molecules regulating muscle oxidative metabolism, fatty acid oxidation, and fast-to-slow muscle fiber type shift. Nevertheless, the ketogenic diet did not change the expression of those factors in this study, suggesting that these factors are not associated with results obtained from the current study. Regarding the search for potential factors, a recent study drew our attention to the muscle fiber type shift by Sema3A. Tatsumi et al. reported increased gene expression of MyHC IIb, but that of slow MyHC was decreased in Sema3A-inhibited myotubes [41]. Moreover, the authors examined muscle regeneration in muscle satellite cell-specific Sema3A knockout mice. After 28 days of cardiotoxin injection, they found that the regenerated muscle exhibited a dramatic increase in type IIb fiber and lower numbers of type I, IIa, and IIx fibers [41]. These findings suggest Sema3A as a negative regulator of type IIb fibers, but as a positive regulator of type I, IIa, and IIx fibers. In light of these findings, we presume that ketogenic diet at least induces muscle phenotype change to more oxidative fiber by enhancing Sema3A in EDL because the ketogenic diet increased Sema3A expression in the present study. Nevertheless, it remains unknown whether Sema3A can modify the expression of mitochondrial and fat oxidation enzymes or mitochondrial biogenesis in skeletal muscle.

Clarifying the precise mechanisms of ketogenic-induced improvements of aerobic metabolism is beyond the scope of this study. Although no change was found in PGC-1α, PPAR-α, and Mef2C in our results, different factors are expected to be associated with those improvements. For example, an earlier study revealed that retinoic acid receptor-related orphan receptor α signaling can regulate CS expression in Hepg2 cells [56]. Moreover, COX-IV expression has been found to be regulated by hypoxia-inducible factor 1 in different mammalian cell types [57]. Furthermore, HAD gene expression was increased in fast-twitch tibialis anterior muscles of leptin receptor disrupted rats, suggesting that leptin negatively regulates HAD expression in skeletal muscle [58]. Importantly, the influences of ketogenic diet on those regulatory systems remain completely unknown at present. Further research must be conducted to elucidate the mechanisms of ketogenic diet-induced improvement of aerobic metabolism.

Despite the improvement of aerobic capacity, the ketogenic diet did not enhance EDL muscle fatigue resistance in this study. Although the reason for this inconsistency cannot be explained, we speculate that a change occurred in the relation between mitochondrial content and function. Mitochondrial content and oxidative capacity are normally correlated. Moreover, increased mitochondrial content and function is important for muscle endurance [59]. However, a recent report of the relevant literature has described mitochondrial respiration as impaired even though the mitochondrial content was increased by a high-fat diet in the red gastrocnemius muscle [60]. Although no ketogenic diet was involved, the results [60] suggest that a higher fat diet disrupts the proportional relation between the volume and the function of mitochondria. Consequently, ketogenic diet-induced improvement of aerobic metabolism might not contribute directly to muscle endurance in our KD animals. Another route of speculation is the experimental conditions under which muscle performance measurements were taken. This study used a standard Krebs–Henseleit solution for an environment of electrical stimulation [45, 46]. However, our KD animals were found to have greater concentrations of circulating ketone bodies, which can be an additional energy source. Moreover, our KD animals showed biochemical adaptation to the ketogenic diet environment. Accordingly, we might observe a change in muscle performance if the experimental solution includes a practical amount of ketone body.

Results of earlier studies also show that the ketogenic diet did not impair the twitch or tetanic force. Although no study using isolated muscle has been conducted, a few studies have examined the relation between the ketogenic diet and muscle strength *in vivo*. Beckett et al. reported that a ketogenic diet fed for four weeks did not affect muscle grip strength in male mice [26]. This trend was also reported for aged mice (12 months of age) consuming a ketogenic diet for a month [6]. The grip strength measurement is used to estimate the maximum limb strength. Therefore, our results support those studies at the isolated organ level. Taken together, our results indicate that a four-week ketogenic diet had no adverse effect on EDL muscle performance.

Finally, the results of this study indicate that the ketogenic diet did not change the muscle mass. The ketogenic diet decreased the body mass gain. Therefore, the relative muscle mass was higher in KD animals. This finding is consistent with earlier reports describing four-week ketogenic diet feeding [6, 24, 25]. Consequently, application of a ketogenic diet for four weeks is not harmful to muscle quantity.

In conclusion, the results of this study demonstrate that a four-week ketogenic diet alters skeletal muscle toward aerobic energy phenotype without obstructing muscle contractile performance in sedentary male rats. Sema3A might partially mediate the phenotypic change. Regarded in perspective, our findings suggest ergogenic benefits of a ketogenic diet for people, such as athletes, who must reconcile body weight loss and muscle performance.

## Supporting information

S1 Raw images (PDF)
