# Supplementary material for: Ketogenic diet feeding improves aerobic metabolism property in extensor digitorum longus muscle of sedentary male rats
Source: PLoS One. 2020 Oct 30;15(10):e0241382. doi: 10.1371/journal.pone.0241382 (PMC7598508; doi:10.1371/journal.pone.0241382)
Supplement: S1 Raw images — (PDF) [file pone.0241382.s001.pdf]

Bands for Fig.2

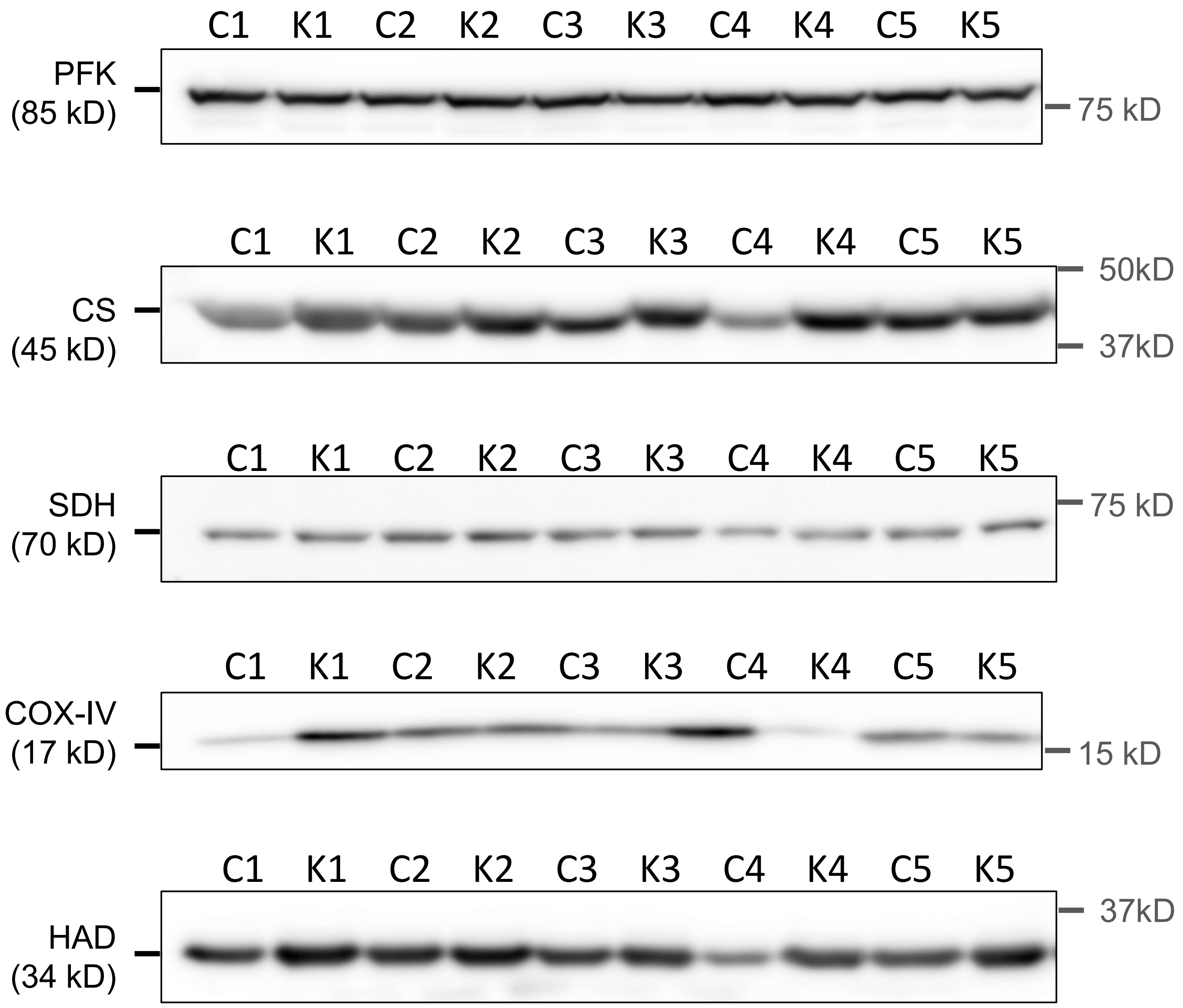

C indicates Control diet group.
K indicates Ketogenic diet group.

Gel images for Fig.3

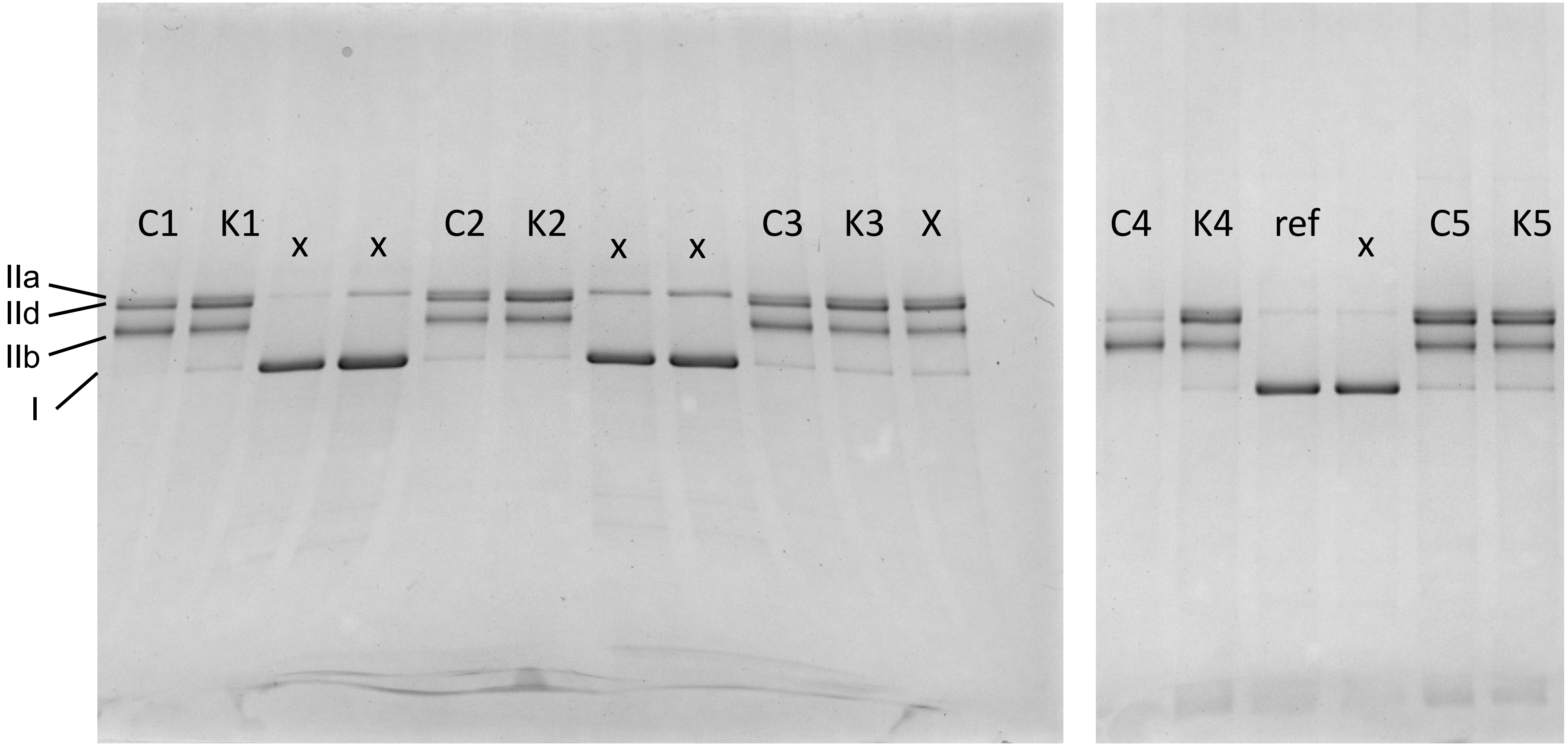

C indicates Control diet group.
K indicates Ketogenic diet group.
ref indicates reference bands.
x indicates un-related test sample for this manuscript.

Bands for Fig.3

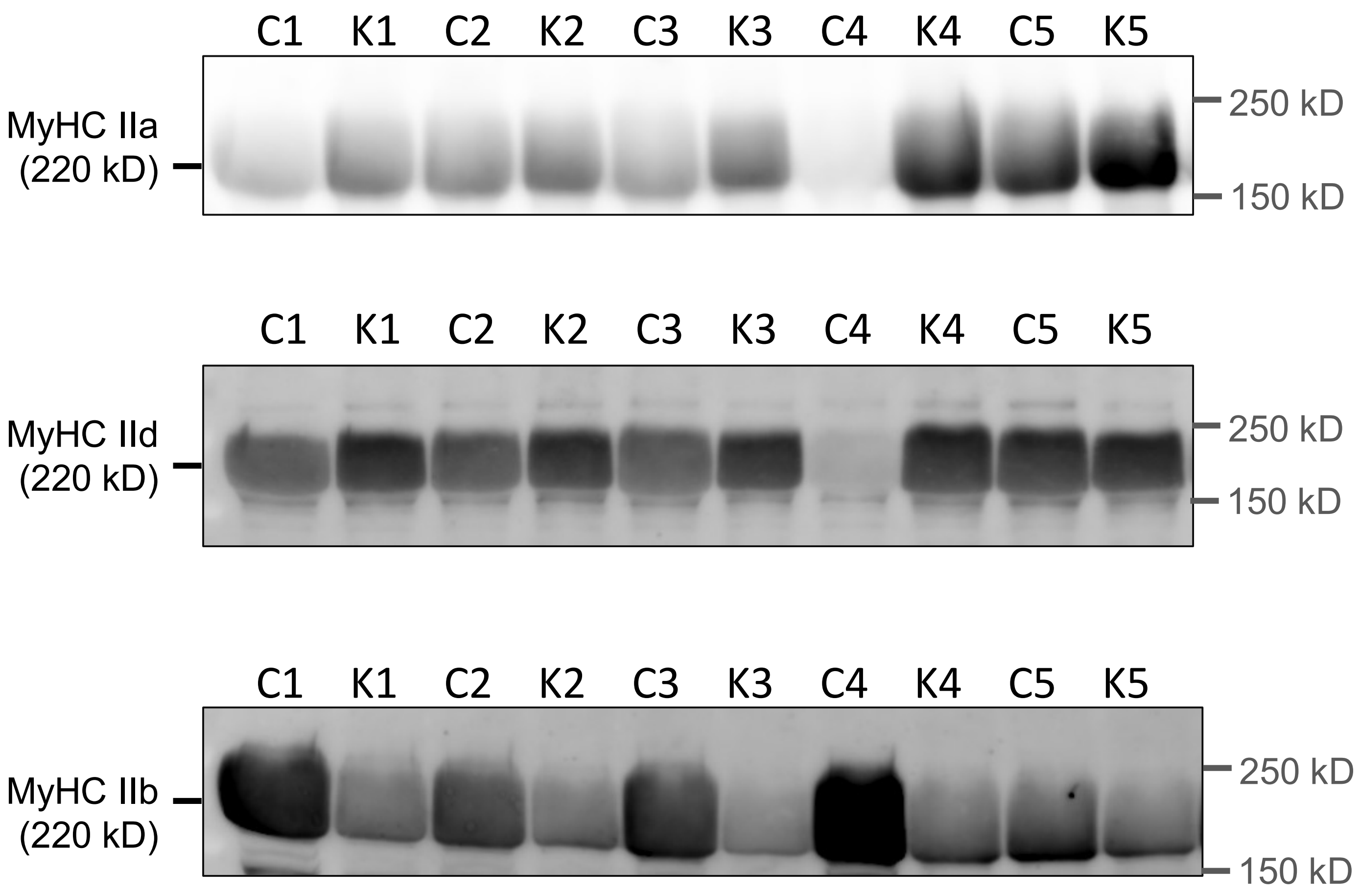

C indicates Control diet group.
K indicates Ketogenic diet group.

Bands for Fig.4 and Fig.5

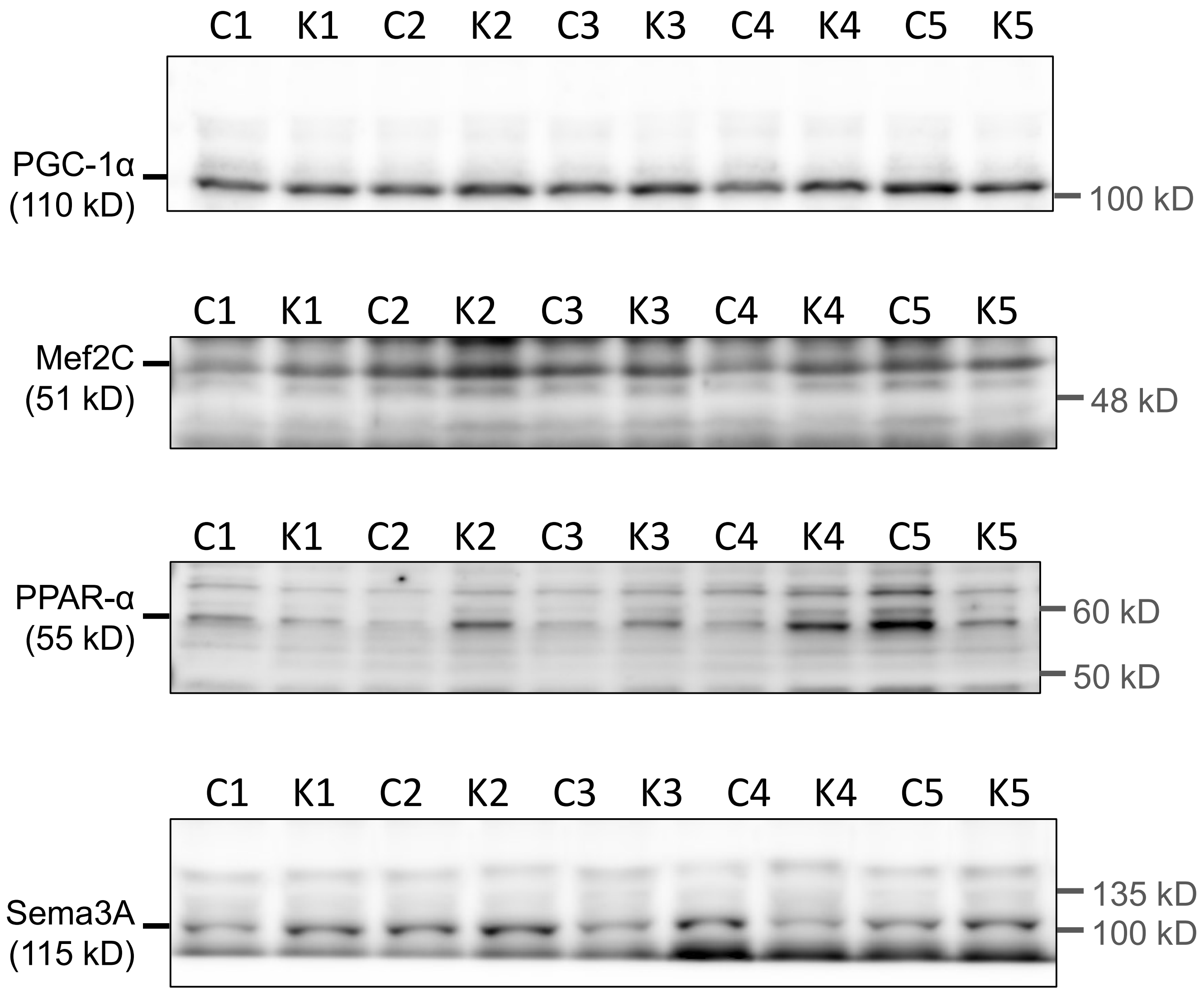

C indicates Control diet group.
K indicates Ketogenic diet group.

Ponceau S stain for PFK

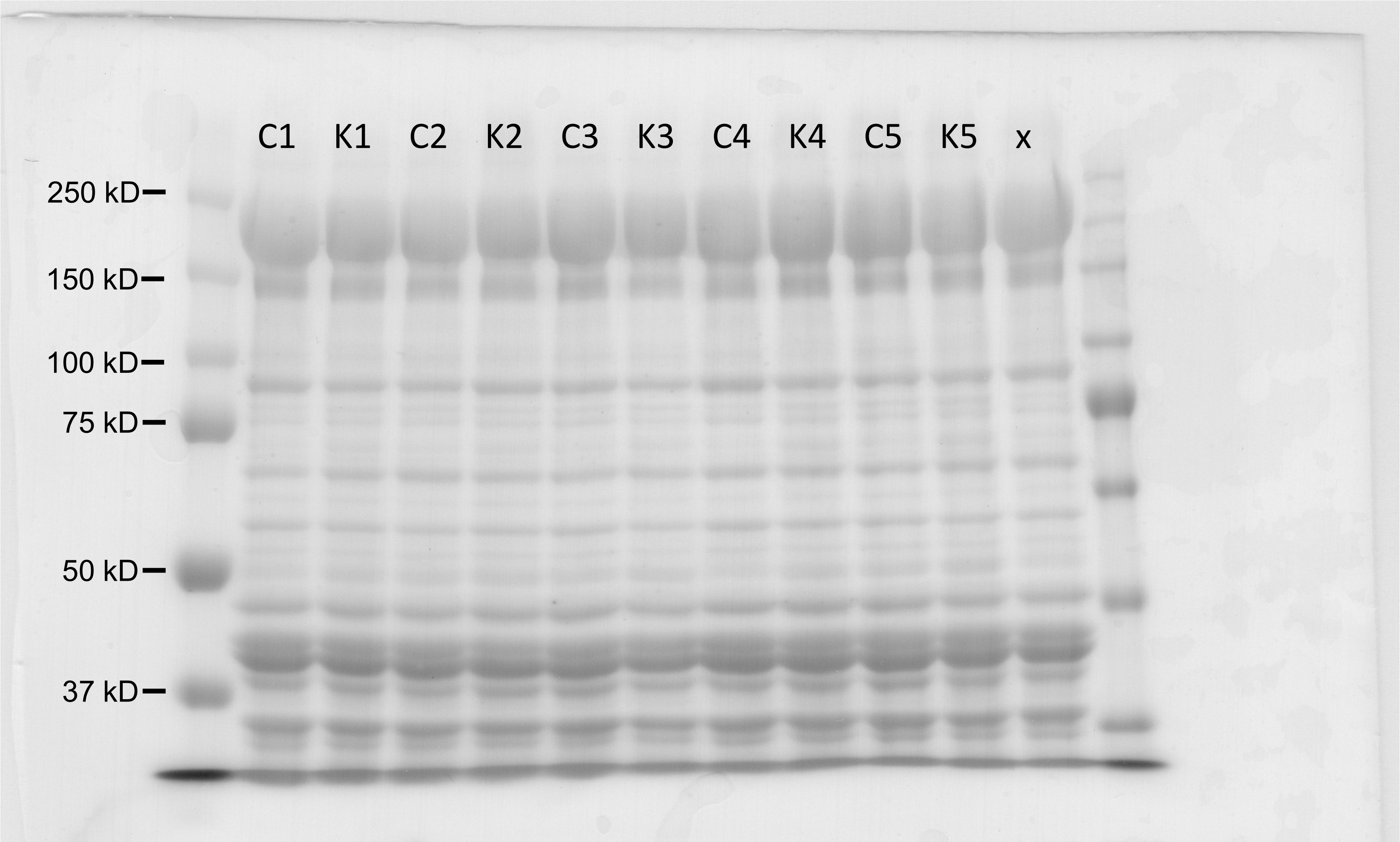

C indicates Control diet group.
K indicates Ketogenic diet group.
x indicates un-related sample for this manuscript.

Ponceau S stain for SDH, HAD

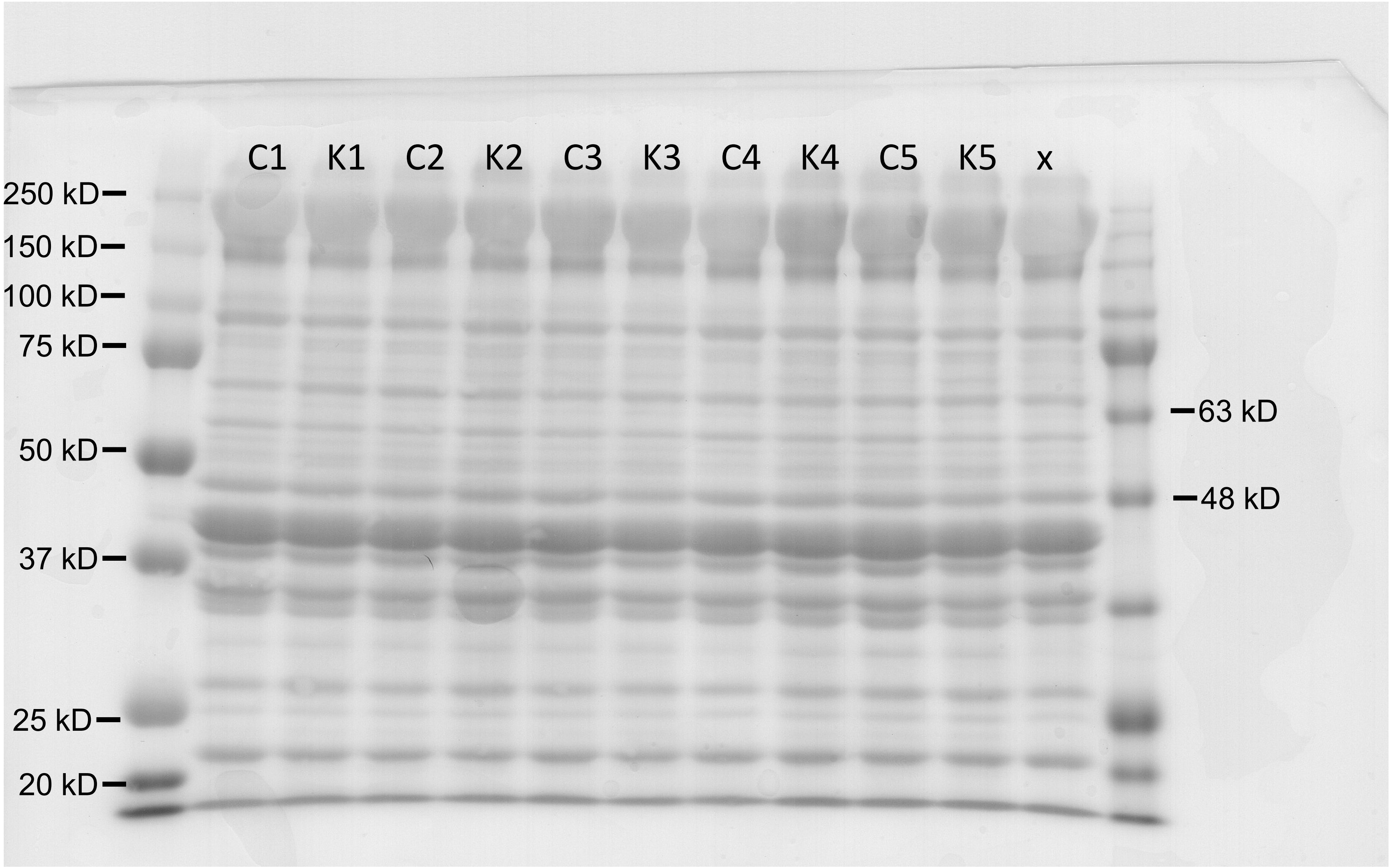

C indicates Control diet group.
K indicates Ketogenic diet group.
x indicates un-related sample for this manuscript.

Ponceau S stain for PGC-1α, COX-IV

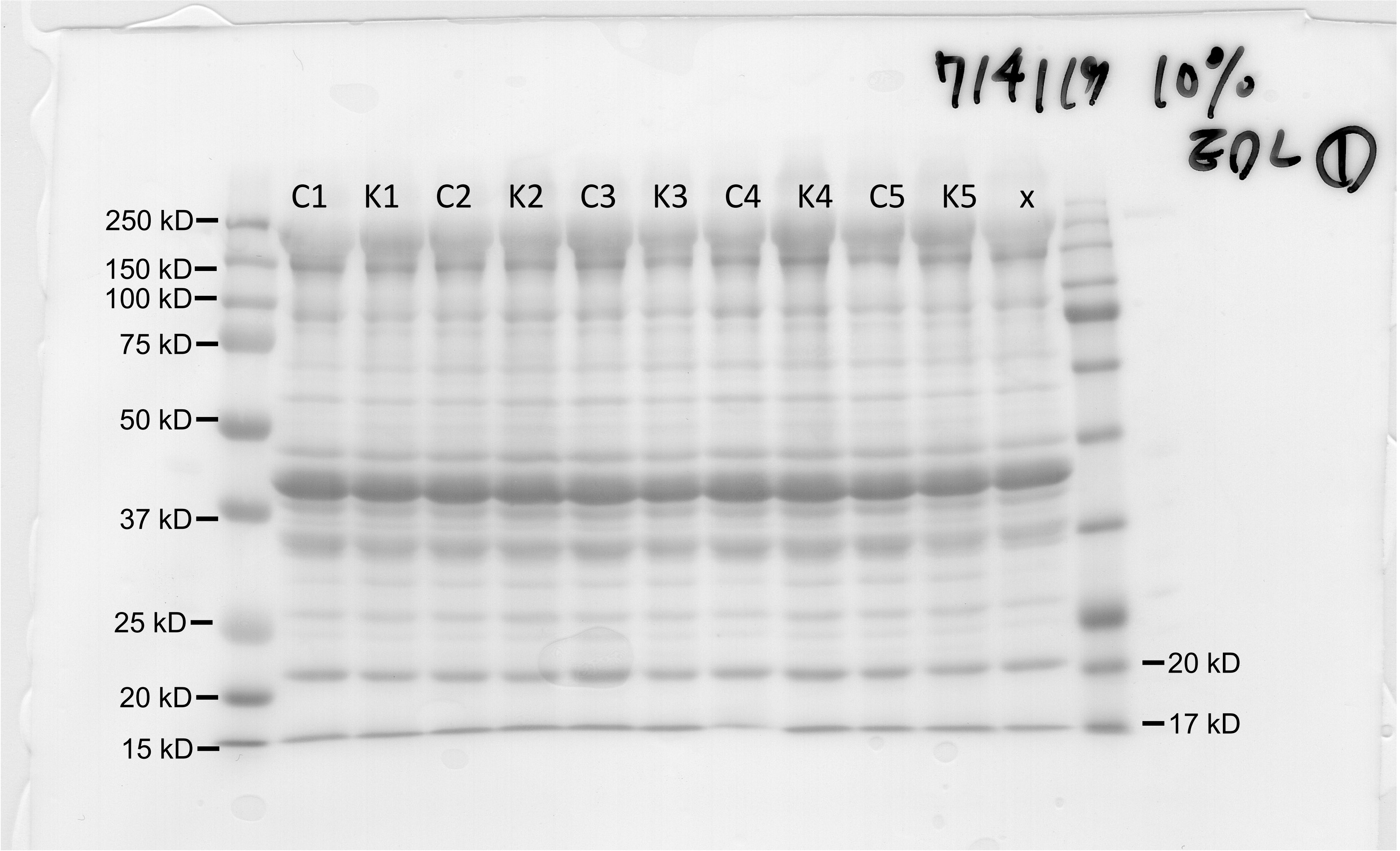

C indicates Control diet group.
K indicates Ketogenic diet group.
x indicates un-related sample for this manuscript.

Ponceau S stain for MyHC IIa

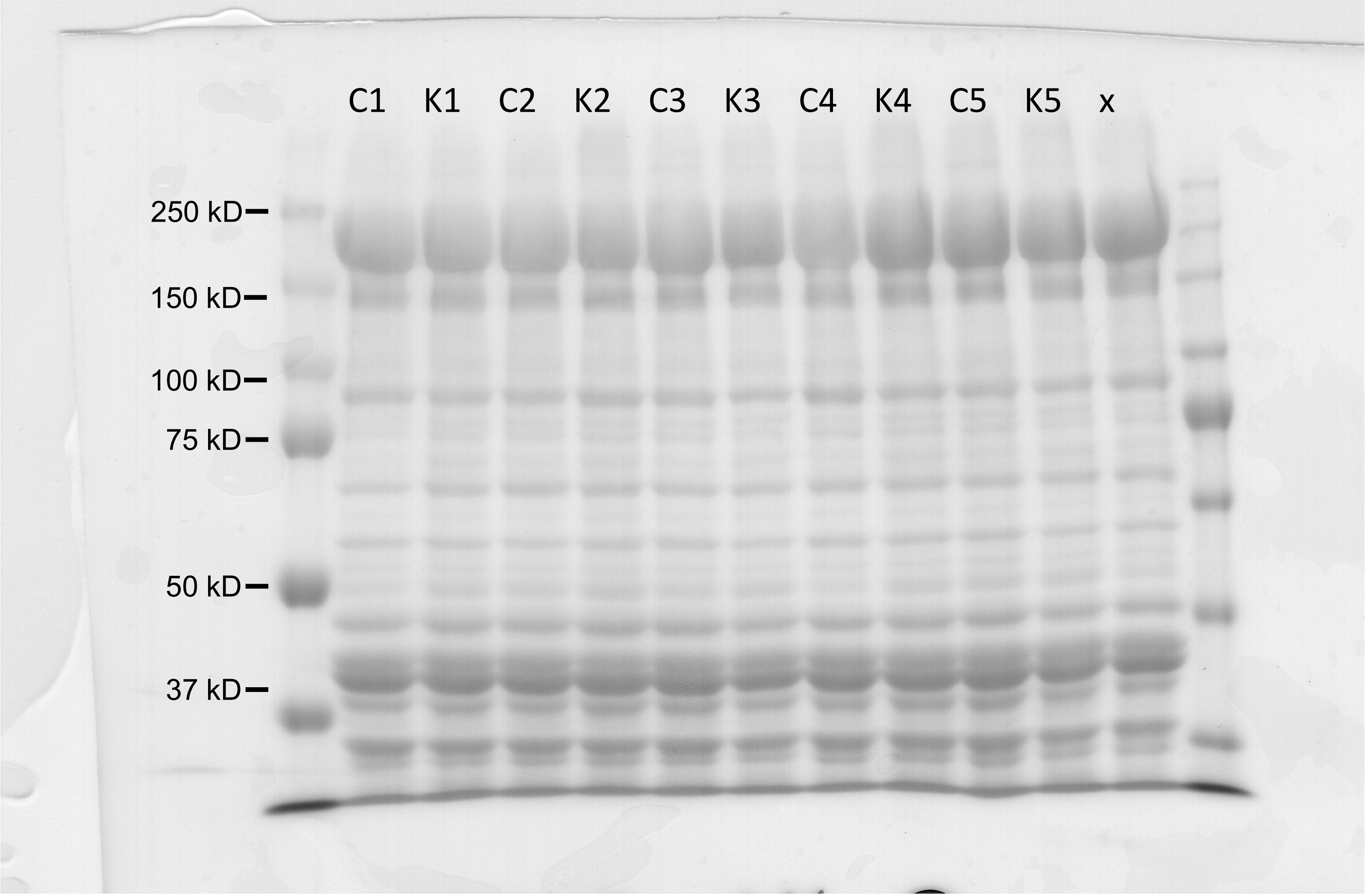

C indicates Control diet group.
K indicates Ketogenic diet group.
x indicates un-related sample for this manuscript.

Ponceau S stain for PPAR-α, Mef2c

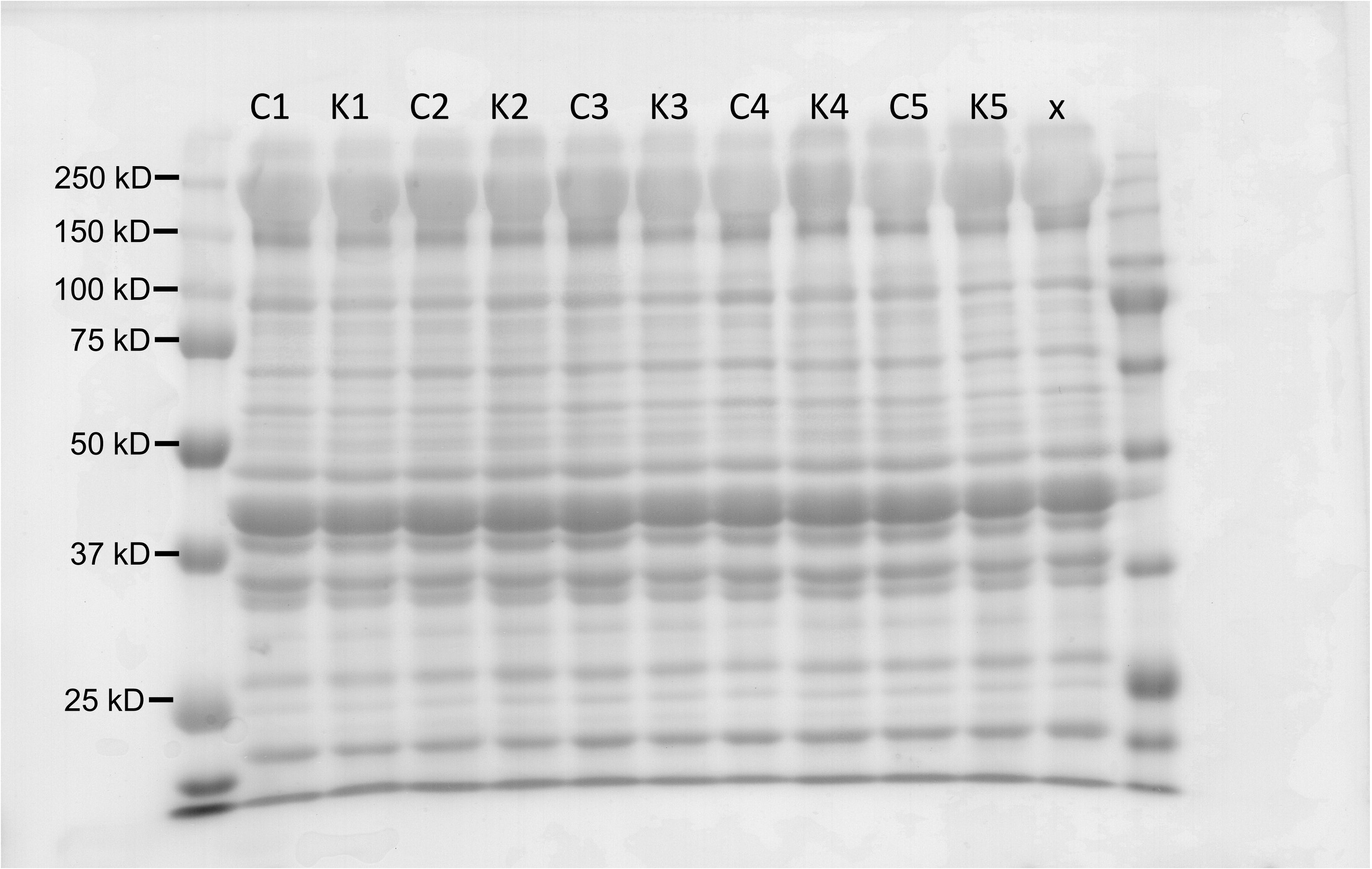

C indicates Control diet group.
K indicates Ketogenic diet group.
x indicates un-related sample for this manuscript.

Ponceau S stain for MyHC IIb

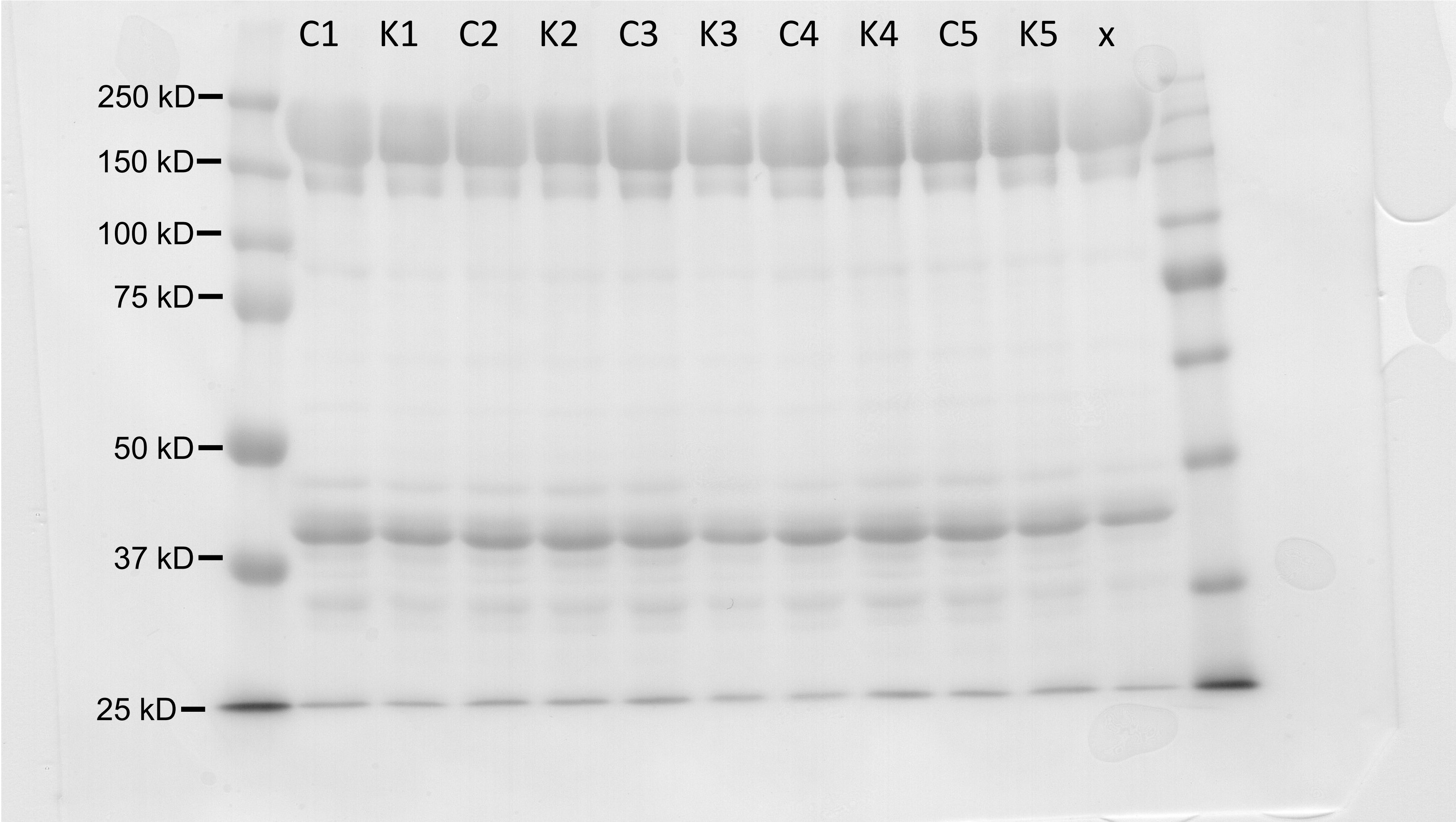

C indicates Control diet group.
K indicates Ketogenic diet group.
x indicates un-related sample for this manuscript.

Ponceau S stain for CS and Sema3A

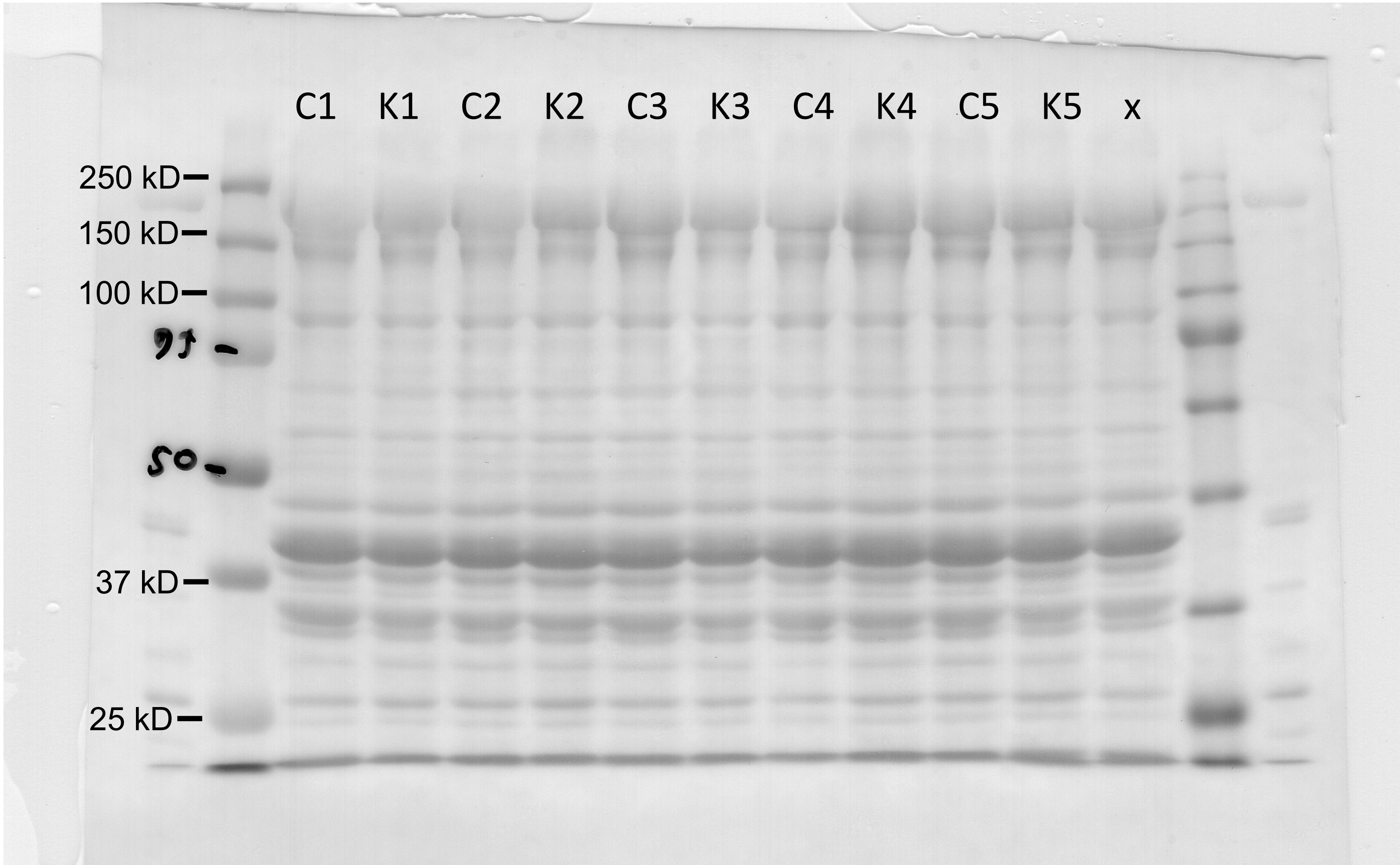

C indicates Control diet group.
K indicates Ketogenic diet group.
x indicates un-related sample for this manuscript.

Ponceau S stain for Mef2c and MyHC IId

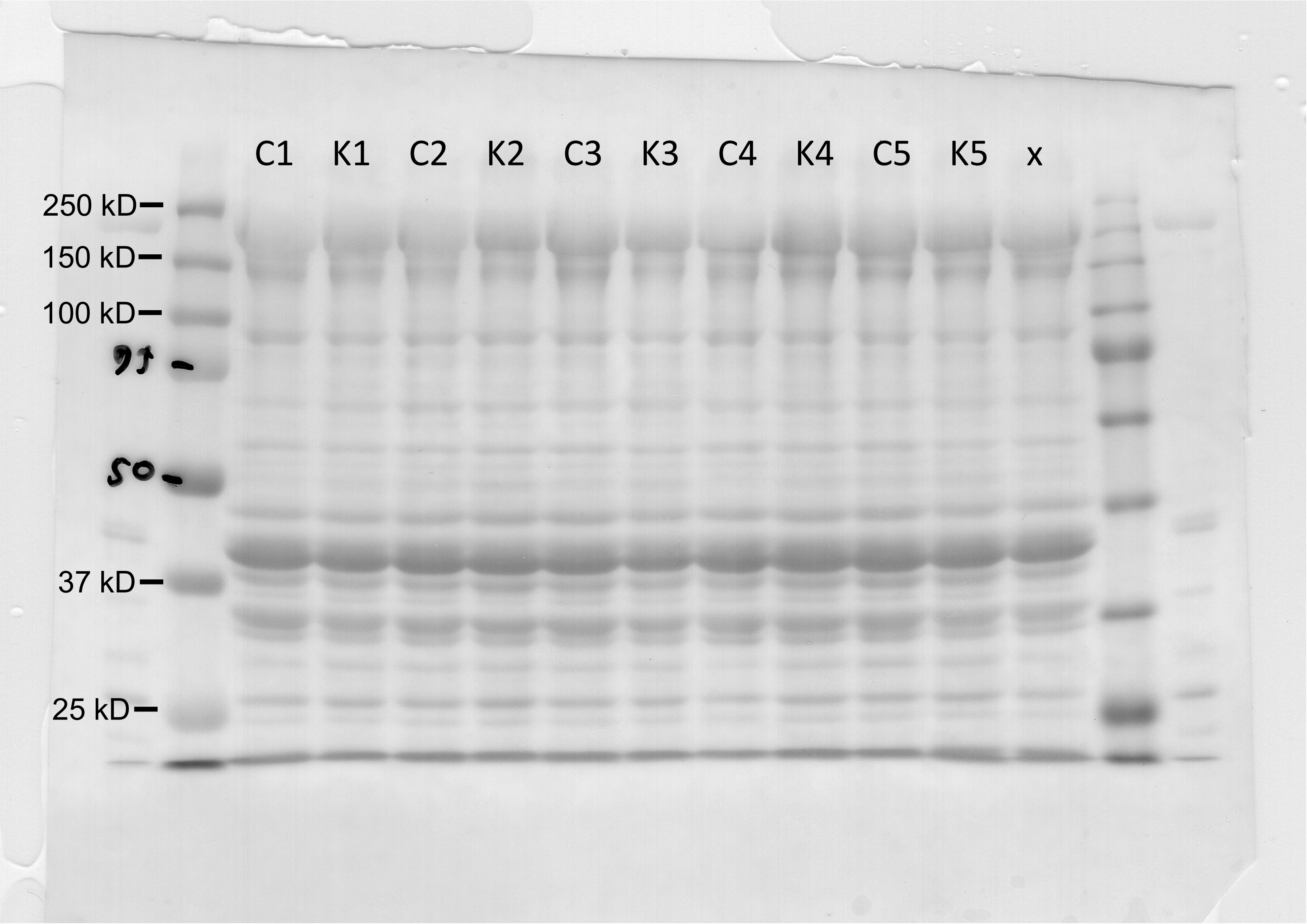

C indicates Control diet group.
K indicates Ketogenic diet group.
x indicates un-related sample for this manuscript.
